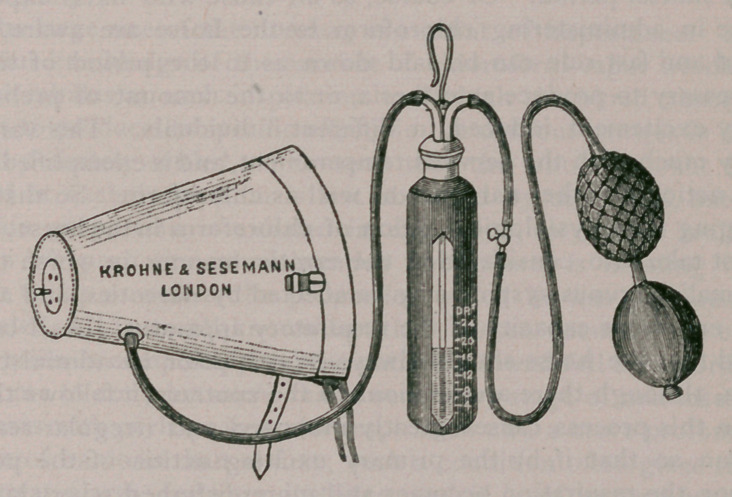# An Improved Apparatus for the Administration of Chloroform to Horses, with Remarks Thereon

**Published:** 1895-10

**Authors:** E. Wallis Hoare

**Affiliations:** Cork


### AN IMPROVED APPARATUS FOR THE ADMINISTRATION OF CHLOROFORM TO HORSES, WITH REMARKS THEREON.

In drawing the attention of the profession to an improved method of administering chloroform to the horse, I do not wish to claim any originality in the principle on which it is founded, viz., that of diluting the chloroform vapor with a proper amount of air before causing the patient to inhale it.

The apparatus, which is made by Messrs. Krohne & Sesemann, London, is now brought as near to perfection as possible, is the result of systematic experiments and practical employment in surgery.

The system is easily explained. Instead of pouring a certain amount of chloroform on a sponge, and fixing the latter in a bag to the animal's head, so as to exclude as much air as possible, the new apparatus, by means of a foot bellows, connected by rubber tubing to a graduated bottle containing the chloroform, and another rubber tube connecting the bottle with a capacious nosebag, insures that the vapor of the drug shall be diluted with a certain proportion of air, and so be more easily and more safely inhaled by the animal. The nosebag is provided with an air-regulator beneath, so that the supply of air can be controlled during administration. Perfect control over the chloroform and air is thus obtained.

The bellows is at first worked slowly and steadily so that the vapor is gradually transmitted to the nosebag and inhaled by the animal, with the least possible amount of excitement and struggling. It was taught, and firmly believed, that if chloroform were administered to the horse in this manner, a large amount of the drug would be required and unfavorable results would follow, hence we are cautioned to exclude air as much as possible, and induce anæsthesia quickly by giving plenty of pure chloroform vapor at first. Being skeptical in this matter, and believing that the only way of arriving at correct conclusions is by practical demonstration, and striking out on new lines, I have devoted special attention to this subject, and have now proved conclusively that chloroform should be, and can be, administered to the horse in a similar manner to human beings.

It is irrational in principle and practice to half suffocate an animal while inducing anæsthesia, and many of the objections to the use of this agent and the dangerous symptoms recorded must be ascribed to the practice of rapid anæsthesia combined with semi-asphyxia. If I were asked what are the most essential features in the safe administration of chloroform, I should say, without hesitation: 1. Time. 2. A proper supply of pure air.

I must admit that by the system of administration which I am at present advocating more time is occupied in the production of anæsthesia than by the other method, but a larger amount of the drug is *not* required, and consciousness returns in a shorter period. Of course, as all those who have experience in administering chloroform to the horse are aware, no hard and fast rule can be laid down as to the period of time necessary to produce anæsthesia, or to the amount of preliminary excitement induced, in different individuals. This varies very much with the nervous temperament, and is exemplified by the action of other narcotics as well as chloroform. So that in judging the physiological action of chloroform in the horse, we must take into consideration the erratic manner in which this animal's nervous system may be affected by narcotics, and also the enormous capacity of the respiratory apparatus. As I contend that the horse should always be cast prior to administration, although there are opinions to the contrary, it follows that even this process causes greatly increased and irregular respiration, so that if, by the primary exciting action of the pure vapor, the respiration becomes still more disturbed, a very large amount of the vapor is inhaled at one time, very probably accounting for the fatal results recorded by some practitioners. The object in administering chloroform is to temporarily remove consciousness, and thus to do away with the perceptions of pain. Unfortunately in bringing about this object we cannot avoid causing a certain amount of depression of the vital centres in the medulla, but as the cerebrum is depressed, and its functions temporarily removed, before the medulla is affected to any extent, it follows that our main object should be to limit the amount of the drug inhaled, so as to produce unconsciousness without causing serious depression of the medulla. How can this be brought about in a safe manner? By causing the animal to inhale a certain amount of the vapor, so that the blood shall contain a sufficient proportion to inhibit the function of the cerebrum for the time being. By the apparatus I have described, the amount of the drug can be accurately measured, so that it is impossible to administer an overdose.

When the proper stage of anæsthesia is reached, we can stop working the bellows and we prevent the administration of an excessive amount of the drug. As I have frequently pointed out there is no necessity to produce full anæsthesia in the case of short operations, such as castration, etc. The first stage is sufficient in the majority of cases; in long operations, after inducing the proper stage, we can leave the nosebag fixed to the head, with the air-regulator open, so that when necessary the assistant can work the bellows and so continue the anæsthesia with safety.

The large size of the nosebag is a point of importance. As yet our knowledge of the amount of chloroform vapor which a horse inhales at each inspiration and which the blood absorbs so as to produce its specific effects in the nervous centres is not at all definitely settled. I contend that the whole amount inhaled at each inspiration is not absorbed by the blood, but a certain proportion is exhaled in expiration, so that in reality when we push the administration the animal is inhaling the fresh vapor as well as the proportion which he has previously exhaled. It is a well-known fact that it often occurs when our operation is completed, and the patient allowed full air in order to come to consciousness, that a secondary stage of anæsthesia appears with perhaps irregular respiration and a more profound stage of narcosis than at first; such a condition often gives rise to great alarm, and very justly so, and it is of importance to arrive at a conclusion as to its cause and prevention. Evidently it is due to an excess of the chloroform vapor circulating in the nervous centres, depending on too much having been administered. We push the drug too quickly at first, and do not give it time to produce its specific effects; we are too anxious to induce rapid anæsthesia and to commence our operation before time; struggling occurs, and more of the drug is administered to prevent this, with the inevitable result.

How are we to prevent and overcome this? I answer, by taking plenty of time before commencing the operation.

Here we have a capacious reservoir for air and chloroform mixed in the nosebag. We are aware of the exact amount of the drug which has entered the bag, then by lessening the amount of air entering by the regulator we compel the animal to inhale what is in the bag before pushing the drug any further. I have found this an excellent plan; no doubt it takes a longer time, but we have the satisfaction of little or no trouble in the return to consciousness.

If animals could be allowed to sleep off the effects of chloroform after long operations, it would be a very important matter. Whenever possible this should be done, and there is no necessity for active measures to bring about a return to consciousness, so long as respiration is being carried on regularly.

This apparatus is of great value in cases where a skilled assistant is not at hand during the performance of a long operation. It is surprising how easily the animal can be kept under the influence of the anæsthetic by a few minutes' working of the bellows. This is far different from the principle of having an assistant pouring the drug in a sponge for administration, causing the operator anxiety as to the after-effects.

There is no waste of the drug in the new apparatus; when the administration is ceased the chloroform left is still in the bottle for use when necessary. There is no danger of asphyxia, in consequence of the large size of the nosebag, and I am safe in asserting that it would be almost a matter of impossibility to kill a horse with this apparatus in any reasonable period of time.

Here I must direct special attention to an important point, that is, the recognition of the proper stage of anæsthesia.

I have frequently shown that the indications afforded by the eye of the horse are of no value as a guide, as even when a stage of anæsthesia sufficient for any operation is induced the reflex action of the eyelids will still persist. The condition of the limbs, as regards muscular power, I regard as the chief sign, also the loss of sensation on striking the animal firmly on the quarter. Catch hold of either fore or hind limbs, and if there is loss of muscular power to extend them anæsthesia is sufficiently complete.

In the operation of castration the condition of the testicles is a sure sign of the proper stage of anæsthesia, the cremaster has lost its contractile power and the organs are flaccid to the touch.

In operations such as neurotomy, the removal of deep-seated tumors, and in operating in quittors, it is necessary to induce a fuller degree of anæsthesia.

The following are the average periods of time and amounts of the drug required, taken from notes of a large number of cases: For yearlings for the operation of castration, well-bred and in good condition, from ¾ of an ounce to 1 ounce; time, from five to eight minutes to produce anæsthesia; for return to consciousness, eight to ten minutes. In common-bred yearlings of small size ½ an ounce is often all that is required, by allowing them time to thoroughly inhale it. For adults, 1½ to 2 ounces are the average amounts, the time varying from ten to fifteen minutes in some cases.

As an example of the exciting effects of chloroform in some horses, I annex the following notes of a case which recently came under my notice:

A valuable thoroughbred racehorse, four years old, in hard condition, had run in a steeplechase two days previously, and, in consequence of his temper, his owner decided to have him castrated. He was carefully cast, and the chloroform administered by the method I have described. There was little preliminary struggling, but the animal neighed loudly and prolonged. The penis was in a full state of erection, and placed against the abdomen; this continued for some time till unconsciousness was complete. This animal took two ounces, and the time occupied fifteen minutes. He came to beautifully, and never showed the slightest after-effects from the operation or otherwise; in fact, he neighed for food on regaining consciousness and returning to the stable.

I have on innumerable occasions administered chloroform to horses for all kinds of operations without any assistant, and now do not consider the procedure any more serious than that of casting the animals.

In the discussion on Prof. Penberthy's paper on "Pain," at the meeting of the National Veterinary Association, it was stated that involuntary struggling did not occur during the operation of castration with the animal under chloroform.

I distinctly stated, as the result of practical experience, that it did occur when the non-vascular portion of the spermatic cord was divided prior to placing the clamp on the vascular portion. Of course if profound anæsthesia be induced, such an occurrence may not be noticed, but what practitioner would induce such a condition in his patient for a quick operation? The involuntary movement I mentioned occurs, as a rule, in all cases where a proper stage of anæsthesia is induced, and other practitioners agree with me that this involuntary movement may occur not only in castration but also in neurotomy. I have frequently noticed that in making post-mortem examinations of horses, soon after having been destroyed by shooting, that on cutting into the muscles there is a distinct contraction and a quivering motion both seen and felt.

In such cases the cerebrum has been destroyed, as well as the vital centres, and still a certain amount of contraction remains, so that we cannot be surprised at involuntary contraction remaining to a certain extent after anæsthesia has been produced.

In concluding these remarks, it is with feelings of great satisfaction that I notice the rapidity with which the advocates of anæsthetics in veterinary surgery are coming forward. The owners of horses, in nearly every instance, give us encouragement; indeed, I know of some cases where they refuse to allow operations to be performed except under the influence of an anæsthetic.

Up to the present our opponents have not adduced a single tangible argument to support their views, and unfortunately they either absent themselves or remain silent on occasions where an opportunity arises for discussion on the subject.

The £ *s. d.* argument will not stand for a moment in the present day. It is opposed to professional progress, it is antagonistic to scientific surgery, it is contrary to the dictates of humanity.

The opposition to the employment of chloroform must be ascribed to a fear of fatal results, depending on want of experience in its use; in some instances the small amount of extra trouble and time involved is grudged to the unfortunate patients, who are compelled to bear the most excruciating pain, inflicted on them by beings possessed of superior intelligence.

We preach from the text, *Humanitas scientia utilitas* to an intelligent public; but do we practise such high-minded principles as are expressed in these words?

Let us clearly demonstrate to our medical *confrères* that we are just as anxious to improve our knowledge in that important adjunct to surgery, viz., the study of anæsthetics, as they are ever striving to do, and that we shall not merit that taunt which was thrown out at one time in a medical journal, viz., that the cruelties attributed to vivisection could not be compared to those which were perpetrated on the lower animals by severe and painful operations performed without the use of anæsthetics.—E. WALLIS HOARE, F.R.C.V.S., *Cork*, in *The Veterinary Record.*

## Figures and Tables

**Figure f1:**
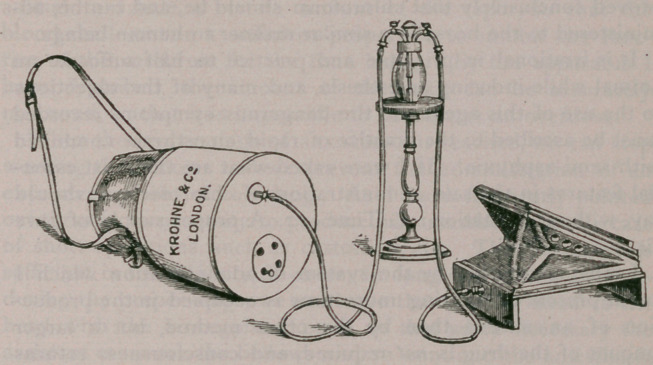


**Figure f2:**